# 

**Published:** 1892-05

**Authors:** 


### AMERICAN MEDICAL ASSOCIATION.

The American Medical Association meets in the city of ~~Chicago~~ on the 7th to the 10th of June, and matters of great practical interest to the profession will come before this meeting.

The several sections will each hold two sessions a day for scientific work, while there is but one session daily of the whole body for hearing the general addresses and the transaction of business.

Dr. J. McFadden Gaston of this city is the chairman of the section of Surgery and Anatomy and his opening address will be upon "Surgery of the gall-bladder and ducts." This will be followed by other papers on gall-bladder surgery, with a discussion of them by those who are familiar with the subject.

The contributions on intestinal and abdominal surgery, with their discussions, will occupy the second day, and the third and fourth days will be taken up with miscellaneous papers on a variety of subjects of great practical interest.

There are already in the hands of the secretary of the surgical section, Dr. F. W. Mason, of Detroit, about forty titles of papers to be introduced into the program, which will be soon announced through the Journal of the American Medical Association.

It is understood that the committee upon improvement of the sections will meet in the city of Detroit on June 6th, the day prior to the opening of the Association.

